# Adjuvant immunotherapy recommendations for stage III melanoma: physician and nurse interviews

**DOI:** 10.1186/s12885-021-08752-1

**Published:** 2021-09-10

**Authors:** Ann Livingstone, Kathy Dempsey, Martin R. Stockler, Kirsten Howard, Georgina V. Long, Matteo S. Carlino, Alexander M. Menzies, Rachael L. Morton

**Affiliations:** 1 NHMRC Clinical Trials Centre, The University of Sydney, Sydney, NSW Australia; 2 School of Public Health, Faculty of Medicine and Health, The University of Sydney, Sydney, NSW Australia; 3 Central Clinical School, Faculty of Medicine and Health, The University of Sydney, Sydney, NSW Australia; 4 Melanoma Institute Australia, The University of Sydney, Sydney, NSW Australia; 5 Faculty of Medicine and Health, The University of Sydney, Sydney, NSW Australia; 6 Department of Medical Oncology, Royal North Shore and Mater Hospitals, Sydney, NSW Australia; 7 Crown Princess Mary Cancer Centre, Westmead Hospital, Westmead, NSW Australia

**Keywords:** Melanoma, Immunotherapy, Interviews, Physicians, Surgeons, Nurse clinicians, Professional role, Decision making

## Abstract

**Background:**

Adjuvant immunotherapy is revolutionising care for patients with resected stage III and IV melanoma. However, immunotherapy may be associated with toxicity, making treatment decisions complicated. This study aimed to identify factors physicians and nurses considered regarding adjuvant immunotherapy for melanoma.

**Methods:**

In-depth interviews were conducted with physicians (medical oncologists, surgeons and dermatologists) and nurses managing patients with resected stage III melanoma at three Australian tertiary melanoma centres between July 2019 and March 2020. Factors considered regarding adjuvant immunotherapy were explored. Recruitment continued until data saturation and thematic analysis was undertaken.

**Results:**

Twenty-five physicians and nurses, aged 28–68 years, 60% females, including eleven (44%) medical oncologists, eight (32%) surgeons, five (20%) nurses, and one (4%) dermatologist were interviewed. Over half the sample managed five or more new resected stage III patients per month who could be eligible for adjuvant immunotherapy. Three themes about adjuvant immunotherapy recommendations emerged: [1] clinical and patient factors, [2] treatment information provision, and [3] individual physician/nurse factors. Melanoma sub-stage and an individual patient’s therapy risk/benefit profile were primary considerations. Secondary factors included uncertainty about adjuvant immunotherapy’s effectiveness and their views about treatment burden patients might consider acceptable.

**Conclusions:**

Patients’ disease sub-stage and their treatment risk versus benefit drove the melanoma health care professionals’ adjuvant immunotherapy endorsement. Findings clarify clinician preferences and values, aiding clinical communication with patients and facilitating clinical decision-making about management options for resected stage III melanoma.

**Supplementary Information:**

The online version contains supplementary material available at 10.1186/s12885-021-08752-1.

## Background

Globally, Australia has the highest melanoma incidence, the deadliest form of skin cancer [1]. Treatment options for patients with resected stage III melanoma include observation (i.e., monitoring and treating if the melanoma recurs), adjuvant immunotherapy, and those with BRAF mutant melanoma adjuvant BRAF-targeted therapy. Randomised trials of adjuvant immunotherapies (e.g. anti-PD-1 antibodies, nivolumab and pembrolizumab) have substantially reduced the risk of melanoma recurrence [2, 3]. Without adjuvant treatment, 10-year survival for patients with resected stage IIIA-D melanoma is between 24 and 88%, worsening by sub-stage, meaning absolute treatment benefits differ [4]. Given these reductions in melanoma recurrence risk, the Australian Government recently approved subsidies for adjuvant nivolumab and pembrolizumab for resected stage III disease [5, 6], as is the case in the United States and Europe [7, 8].

Trade-offs between treatment harms, such as toxicities and cost, and therapy benefits need to be considered relative to the alternative option of observation. Furthermore, immunotherapy can cause life-threatening conditions, such as myocarditis or colitis; however, unlike BRAF-targeted therapy, immunotherapy can cause permanent toxicities, including endocrinopathies (thyroid, pituitary, pancreatic) [2, 3]. Additionally, data are awaited regarding whether adjuvant immunotherapy immediately after resection versus later after recurrence leads to improved survival [2]. In the patient context of no ‘active’ melanoma, these scenarios complicate clinical adjuvant immunotherapy recommendations.

Clinical recommendations regarding adjuvant immunotherapy present unique challenges. Uncertainty about the optimal timing of treatment, e.g. following resection or when recurrence occurs, must be considered alongside treatment risks, including mild, fatal and irreversible toxicities. Despite a recent review of physician and nurses immunotherapy preferences [9], gaps in the current literature exist. In addition, research limitations surround physician and nurse recommendations for adjuvant immunotherapy, including sparse evidence on how decisions regarding patients with stage III melanoma are made and a lack of qualitative research exploring clinical issues. Given the additional immunotherapy risk of lifelong toxicity compared to BRAF-targeted therapy, it is imperative to understand how health care professionals trade-off the potential immunotherapy harms and benefits. This study aimed to explore the factors physicians and nurses considered when making adjuvant immunotherapy recommendations for patients with resected stage III melanoma. Factors will assist health care professionals and patient communication regarding management options. Further research plans to investigate treatment trade-offs, facilitating a deeper understanding of adjuvant treatment clinical decision-making and how different physicians value outcomes.

## Methods

### Recruitment and setting

Prior research with patients with resected stage III melanoma and their partners acknowledged the potential for all melanoma clinicians (medical oncologists, surgeons, dermatologists and nurses) to influence their patients’ adjuvant immunotherapy decision-making [10]. As such, purposive sampling [11] enabled the recruitment of melanoma physicians (medical oncologists, surgeons and dermatologists) and nurses with experience treating patients with resected stage III melanoma from three Australian tertiary melanoma centres. Melanoma nurses were clinical nurse consultants providing direct patient care, monitoring treatment toxicities, providing treatment information and psychological support or clinical trials managers coordinating patient recruitment to medical, surgical, and radiation clinical trials. Participants were able to provide informed consent and complete the interview without an interpreter.

### Interview process

The research team included a lead researcher, a health economics PhD candidate (AL), three social scientists (KD, KH and RLM), and four medical oncologists (AMM, GVL, MRS, MSC), three of whom were interviewed. One author (AL) facilitated an individual, face-to-face or telephone interview between July 2019 and March 2020. Interview topic lists were compiled based on a systematic review identifying factors melanoma clinicians considered about immunotherapy; and in-depth discussion with investigators drawing on their clinical knowledge and experience [9]. A semi-structured interview guide facilitated discussion about the factors physicians and nurses considered when recommending adjuvant immunotherapy for resected stage III melanoma (Additional file 1). Most interviews were completed before the recent Australian government reimbursement of adjuvant nivolumab or pembrolizumab for resected stage IIIB-D melanoma. The median interview duration was 42 min (range 22–75), and interviews were audio-recorded and transcribed verbatim by a professional transcribing service. The Sydney Local Health District Human Research Ethics Committee (HREC/18/RPAH/736) granted ethics approval. Participants provided informed written consent, with the Declaration of Helsinki underpinning this study [12].

### Qualitative approach and data analysis

This qualitative study used an interpretative approach. Consideration of the participants’ beliefs, meanings, perceptions and underlying motivations when recommending adjuvant immunotherapy were explored [13]. Given that physician decision-making was the main focus, expected utility theory was drawn upon, meaning choices are rational and aim to maximise utility for most patients [14]. Data were analysed using thematic analysis, and participants were recruited until data saturation. Saturation was confirmed when no new themes emerged despite continued data collection [15]. HyperResearch software (Researchware, Massachusetts) was used to manage de-identified manuscripts. Transparent reporting was assured using The Standards for Reporting Qualitative Research checklist (Additional file 2) [16]. AL generated the initial codes, using her familiarisation with the data. RLM and AL discussed the codes, and an inductive and iterative data review was undertaken. KD independently reviewed a sample of data and generated a list of codes. All codes were then discussed by AL, RM and KD, refined into main topics and reorganised into sub-themes and themes, resulting in an agreed coding framework. Physician and nurse quotes illustrate sub-themes and themes (Table 2). Peer debriefing and validation by co-authors addressed researcher subjectivity and biases [17].

## Results

**Fig. 1:**
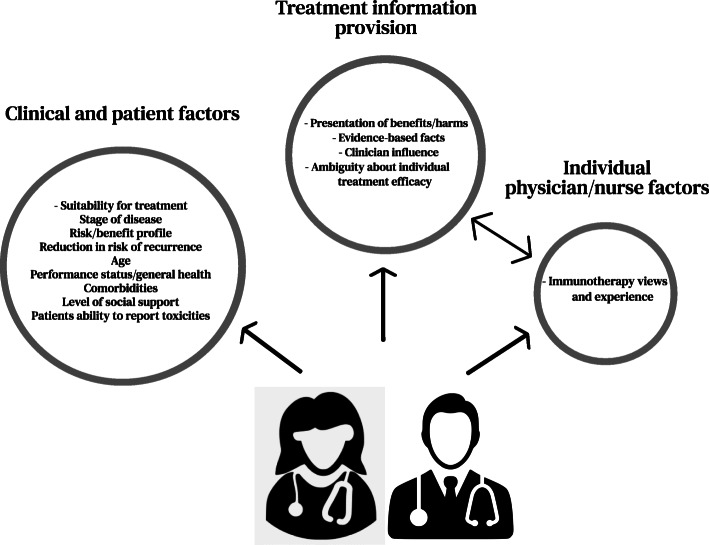
Physician and nurse views about recommending adjuvant immunotherapy: themes and sub-themes. Themes discussed more frequently are displayed as larger circles. Bi-directional arrows refer to the relationship between themes

Twenty-five physicians and nurses, aged 28–68 years, 60% females, including eleven (44%) medical oncologists (three trainees), eight (32%) surgeons, five (20%) nurses, four clinical nurse consultants, one clinical trials manager, and one (4%) dermatologist, attended a single interview. The majority (66%) were recruited from a specialist melanoma centre. Over half the sample managed five or more new resected stage III patients per month who could be eligible for adjuvant immunotherapy. Most participants (80%) considered adjuvant immunotherapy trials for their patients with stage II melanoma (Table 1). A standardised questionnaire collected sociodemographic data (Additional file 1). Three themes about recommending adjuvant immunotherapy emerged: [1] clinical and patient factors, [2] treatment information provision, and [3] individual physician/nurse factors (Fig. 1). Each theme contained up to four sub-themes, with supportive participant quotations presented in Table 2.

**Table 1:** Physician and nurse characteristics

Characteristic, n (%)	Physicians/Nurses (***n*** = 25)
*Sex*	
Male	10 (40%)
Female	15 (60%)
*Age, years*	
Median (range)	44 (28–68)
< 40	10 (40%)
40–60	12 (48%)
> 60	3 (12%)
*Study centres*	
Melanoma Centre 1	14 (56%)
Melanoma Centre 2	3 (12%)
Melanoma Centre 3	8 (32%)
*Healthcare speciality*	
Medical Oncologist^a^	11 (44%)
Surgeon	8 (32%)
Dermatologist	1 (4%)
Clinical Nurse Consultant/Clinical Trials Manager^b^	5 (20%)
*Hours worked per week*	
< 50	8 (32%)
50–60	16 (64%)
> 60	1 (4%)
*Years since completing specialist medical/nursing training*	
< 10	11 (44%)
10–20	8 (32%)
> 20	6 (24%)
*Years experience treating melanoma*	
< 10	9 (36%)
10–20	11 (44%)
> 20	5 (20%)
*% of patients seen with melanoma*	
< 50	6 (24%)
50–75	5 (20%)
> 75	14 (56%)
*Average number of stage III patients eligible for adjuvant immunotherapy seen per month*	
< 5	12 (48%)
5–15	11 (44%)
> 15	2 (8%)
*% of physicians and nurses by speciality recommending immunotherapy clinical trials for stage II patients*^*c*^	
Medical Oncologist^a^	9 (36%)
Surgeon	6 (24%)
Dermatologist	1 (4%)
Clinical Nurse Consultant	4 (16%)

^a^Three respondents were medical oncology trainees currently completing specialist training

^b^One respondent of five was a Clinical Trials Manager

^c^80% of participants recommended adjuvant immunotherapy trials for patients with stage II melanoma

**Table 2:** Physician and nurse themes, sub-themes and key quotes

Theme	Sub-theme	Physicians and nurses quotes
1. Clinical and patient factors (stage of the disease, treatment risk/benefit profile, the potential reduction in the risk of recurrence, age, performance status/ general health, comorbidities, level of social support, and the patients’ ability to report treatment toxicities)		1. *“If people have IIIC or IIID disease, I’m probably a bit stronger with encouraging them to have adjuvant (immuno)therapy.” Medical Oncologist, participant #5* 2. *“The people (patients) with the higher stages of stage III (melanoma) should be prioritised (for immunotherapy); we know there are statistically significant differences (patient outcomes are poorer with higher stage melanoma).” Surgeon, #7* 3. *“I think (adjuvant immunotherapy should be discussed with) every patient who has stage III disease..unless they’re 90 and incoherent, they should be seeing a medical oncologist, it’s only a scan or a symptom away, and they’re Stage IV.” Surgeon, #9* 4. *“Particularly young people I think can feel a bit superhero … just cut it out (melanoma), and I’ll be fine” Medical Oncologist, #2* 5. *“I think people who can tolerate it, people who are in good health, people with the greatest potential absolute benefit, people with the most to lose in terms of life, so younger people you’d be encouraging those people more than people in their late 80s. I think it’s a similar conversation with everyone; it’s more to determine whether it’s right for them. I think there’s a natural tendency that younger people with worse disease would want to have it.” Surgeon, #12* 6. *“I’d certainly look at life expectancy from comorbidities and factor that into a recommendation. In the extreme, someone who has a chronic disease with limited life expectancy and is living in a nursing home … I suggest that adjuvant therapy is not going to offer very much.” Medical Oncologist, #1* 7. *“Patient awareness to handle, manage, report (immunotherapy) toxicities promptly is very important. I would not be keen to give adjuvant immunotherapy to a patient who was not reliable in terms of engaging & communicating with the treating team.” Medical Oncologist, #2* 8. *“You could have a Sydney-based patient who just doesn’t communicate and doesn’t tell you when things are potentially problematic; maybe actually location isn’t necessarily what the issue is, maybe it’s more about (a lack of) communication.” Surgeon, #9*
2. Treatment information provision	i. Presentation of benefits and harms	*1. “There is a group of patients where I don’t feel that uncomfortable about not offering them drug (immunotherapy) because it isn’t just the amount of benefit you think they’ll get for the risk of side effects. You’re probably much nearer to equipoise with the risks and benefits.” Medical Oncologist, #4* *2. “I leave a lot of the nitty-gritty discussion up to the medical oncologists. They’re the best people to discuss all of the trial options available, the potential side-effects of treatment, the percentage benefit, and risk; I leave most of that nitty-gritty discussion to them.” Surgeon,#13* *3. “I outline the concept of adjuvant therapy and the types of adjuvant therapy that will be offered, but I try to avoid being too detailed about the risks and benefits. I talk about it in very general terms; I expect the people who are handling the drugs (medical oncologist) to be more specific about the information that is provided.” Surgeon, #7*
	ii. Evidence-based facts	1. *“(Patients) all get written information, they’ll all get verbal information from the doctor and us (Clinical Nurse Consultant, CNC),..and then we’ve also got the videos that we (melanoma centre) made.” CNC, Medical Oncology, #25* 2. *“We (melanoma centre) would highly suggest that’s not standard (care), and if you want to have treatment (immunotherapy), you have to be on the clinical trials. That’s our approach.” Medical Oncologist, #6* ^a^
	iii. Physician/Nurse influence	1. *“I think that people (patients) think through; “Is it (immunotherapy) worth it?” and they think quite a lot (about weighing up the treatment benefits and harms), I think (patients) can be swayed by the impression their clinician gives them about treatment.” CNC, Medical Oncology, #18* 2. *“I don’t want to scare them (the patient) off the idea of immunotherapy, and I want to emphasise the benefit more so than the side effects.” Surgeon, #13* 3. *“The trickiest one is when you don’t think they (the patient) should be treated (with immunotherapy) because they have dementia and are in a nursing home … you can’t see that their quality of life will improve, I’ll go pretty aggressive on not wanting to give them treatment. I certainly wouldn’t give treatment to a patient that will harm them and give them no benefit.” Medical Oncologist, #1*
**Theme**	**Sub-theme**	**Physicians and nurses quotes**
2. Treatment information provision (continued)	iv. Uncertainty about individual treatment efficacy	1. *“We don’t really know if it may be better to prevent it now (treat melanoma with adjuvant immunotherapy after surgery) than to treat it (using immunotherapy) when it happens later (if the melanoma recurs).” Surgeon, #12* 2. *“I had a young lady with stage III disease who’d said no to immunotherapy … she grasped that we didn’t know whether giving adjuvant (treatment) upfront or if it metastasised was better … She said, there’s no evidence, if it’s better treating it when it becomes Stage IV, she’s young, with a young child … she made an informed decision.” Surgeon, #11* 3. *“Some patients struggle with adjuvant (therapy) generally about has the drug (immunotherapy) worked. So there’s always this question at the end of treatment, so am I good now? That’s much easier with advanced cancer..you start a drug, the scan looks better, it’s working, the scan looks worse, it’s not working,..the idea that you might consider it like insurance or improving your odds.” Medical Oncologist, #3*
3. Individual physician/ nurse factors		1. *“The most severe toxicity I had with adjuvant (immunotherapy) was someone I saw as an opinion who pushed very hard for treatment at home,..they had a 6-month hospital admission, they were in ICU for 2 months very unwell, so clearly that person had a strong indication for treatment, but you know they were not far off dying from treatment. I think it does subconsciously alter your threshold for how you discuss the treatment options.” Medical Oncologist, #3* 2. *“Comparing (adjuvant immunotherapy) to other adjuvant treatments like they do in breast cancer, you’ve got 6 months, basically where your life is definitely written off because they will be very ill. With this (immunotherapy), although there is the risk of side effects, it’s a bit more unpredictable; the chances are, you’ll be fine.” Medical Oncologist, #4* 3. *“Immunotherapy is very different to chemotherapy. It is generally much better tolerated than chemotherapy, and I specifically say things like your hair won’t fall out, you’re not going to become very sick and have lots of nausea and vomiting.” Surgeon,#19* 4. *“If the patient is motivated … we (Australia) have compassionate access (to immunotherapy) … we can refer patients to local doctors, and they have this Link program (immunotherapy program enabling infusion delivery in the patients home).” Medical Oncologist, #10*

^a^Physicians’ and nurses’ responses when questioned about their immunotherapy approach for patients with resected stage II melanoma

### Clinical and patient factors

Factors influencing a patient’s suitability for immunotherapy included: the stage of disease, age, performance status/general health, comorbidities (e.g. autoimmune disease), level of social support, and the patient’s ability to promptly report treatment toxicities.

All participants indicated that the patient’s stage of disease was influential for immunotherapy recommendations. Patients with more extensive disease, stage IIIC/D melanoma, were more strongly encouraged to consider treatment than those with earlier stage melanoma, stage IIIA/B. Most participants agreed that all patients with stage III disease should discuss adjuvant immunotherapy. However, due to the lower risk of recurrence in stage IIIA, many were comfortable when this group decided against treatment.

Patient age and performance status were motivating factors for some physicians’ and nurses’ treatment recommendations. Several were less likely to recommend immunotherapy to those over 80 years with low-performance status, particularly if their melanoma recurrence risk was low or comorbidities limited their life expectancy. Some younger patients felt ‘invincible’ and ‘did not have time for treatment’. However, this often resulted in health care professionals increasing the strength of their immunotherapy recommendation for these individuals. Several medical oncologists raised concerns about recommending immunotherapy for patients they considered less likely to promptly report toxicities, consequently reducing the physicians’ ability to manage treatment safely.

### Treatment information provision

Four sub-themes included the presentation of benefits and harms, evidence-based facts, physician/nurse influence, and the uncertainty about individual treatment efficacy. All participants discussed their role in presenting immunotherapy benefits and harms to patients. All stated their treatment recommendations were linked to their perception of the treatment risk/benefit profile and the absolute immunotherapy benefits and harms for each patient, that is, reducing the risk of melanoma recurrence versus the risk of severe toxicities. Surgeon respondents in our cohort commonly discussed treatment benefits and harms in ‘broad’ terms. All surgeons referred their patients to medical oncologists, enabling adjuvant immunotherapy expertise and discussions regarding individual risk/benefit profiles.

Physicians and nurses felt strongly that their role was to provide evidence-based facts to patients to inform their decision-making. All participants were comfortable discussing adjuvant immunotherapy for stage III melanoma, knowing that most patients could access drugs at ‘no-cost to them’ via compassionate use schemes or clinical trials at the time of interview [5, 6]. Adjuvant immunotherapy for stage II melanoma was viewed as experimental, with many participants discussing access to treatment via clinical trials. Several physicians and nurses discussed modes of information delivery to their patients, including written and verbal, videos and website resources, with many favouring a multimodality approach. Commonly, participants noted that health care professionals repeating information were essential to inform and reconfirm each patients’ level of understanding.

Many participants noted that their opinion was influential during patient immunotherapy decision-making. The dermatologist and surgeons were less likely than other physicians and nurses in our sample to discuss their treatment recommendations with patients. Several participants noted that their emphasis on a particular treatment aspect (for example, survival gains versus harms or toxicities) might impact patient decisions. There was some awareness of positive or negative ‘framing’, mainly by the female participants in our sample.

Physicians and nurses verbalised difficulty determining the efficacy of adjuvant treatment in an individual because there was no visible disease to follow, nor is there a specific biomarker to identify benefits from adjuvant therapy. Many reported uncertainties about disease recurrence and whether adjuvant therapy altered the outcome. For example, if the melanoma did not recur following immunotherapy, it may not have recurred without treatment, and conversely, if recurrence did occur, it could have recurred earlier without therapy. Several medical oncologists and most surgeons discussed the dilemma of treating patients prophylactically after resection versus treating those when the melanoma recurred.

### Individual physician/nurse factors

Many participants acknowledged that their personal views and experience might influence patient treatment decisions. Several physicians considered previous patients with poor outcomes, mainly when the circumstances were similar, e.g. the disease stage or the sequence of events. Some considered adjuvant immunotherapy to be less burdensome than adjuvant chemotherapy, with less onerous administration and lower toxicity frequency. Physicians valued the ability to refer patients to competent specialists located in regional centres, reducing rural patients’ need to travel long distances for treatment and follow-up.

## Discussion

Results suggest physicians’ and nurses’ primary concerns when recommending adjuvant immunotherapy to patients with resected stage III melanoma were clinical and patient factors, such as melanoma sub-stage and an individual’s treatment risk/benefit profile. Secondary factors included participants’ uncertainty about adjuvant immunotherapy efficacy and their views about the treatment burden patients might be willing to tolerate. Other patient factors affecting recommendations included comorbidities, general health, ‘older’ age, the degree of social support and the perceived ability to report toxicities promptly.

These findings align with and enhance previous research in other cancers. Melanoma physicians and nurses identified overall and recurrence-free survival and severe immune-related treatment side-effects as highly influential factors in their immunotherapy decision-making [9]. Also, our study identified the patient’s ability to rapidly communicate treatment side-effects, comorbidities, and the treatment risk/benefit profile as critical drivers of clinical decisions in adjuvant stage III disease. Our results confirm physicians and nurses complexities when trading off adjuvant immunotherapy risks and benefits to inform their patients’ treatment recommendations. Prior research confirms melanoma and cancer physicians’ preferences to minimise risks, such as treatment toxicities [18–23]. Moreover, physician and nurse considerations when recommending systemic cancer treatments are not well understood. A recent study highlighted the challenges physicians face with continuing treatment and the potential to cause more harm than good [24].

The terminology used to communicate healthcare information is critical, with patients preferring simple language when melanoma health care professionals explain adjuvant immunotherapy [10]. In turn, this assists with patients’ understanding of treatment and empowers decision-making. All surgeons provided their patients with basic treatment information and referred them to medical oncologists for a detailed discussion regarding an individual’s adjuvant immunotherapy risks and benefits. Optimal communication strategies in cancer care and melanoma are well documented. Earlier studies align with our results, confirming that patients with cancer favour written and audio information in addition to physician and nurse discussion [25–27].

In our sample, many medical oncologists and surgeons voiced uncertainties regarding the optimal timing of immunotherapy. Physicians’ and nurses’ opinions differ; however, the lack of clarity is compounded due to the dearth of evidence regarding best practice. Considerations included the option to treat adjuvantly following surgery or monitor and treat if the melanoma recurs. Currently, it is unknown whether treating patients earlier following resection, e.g. adjuvantly or later after recurrence, improves patient outcomes. Resected melanoma patients receiving a placebo in the adjuvant pembrolizumab versus placebo trial were eligible to crossover if recurrence occurred; results when available may assist in answering this question [2].

Arguably, individual physician and nurse immunotherapy views and experiences influenced recommendations. In addition, the level of treatment experience varies across medical specialties. Results demonstrate that different specialists were more or less likely to discuss their treatment recommendations with patients. For example, medical oncologists drew on their expertise in managing adjuvant therapy, whereas the dermatologist and surgeons were less inclined to discuss their treatment recommendations with patients. Physicians and nurses with more adjuvant immunotherapy experience were less likely to be impacted by adverse treatment effects; conversely, those with less experience may have a ‘skewed’ view of reality. However, patients are managed by multi-disciplinary teams, and the impact of other specialists’ advice on patients choices cannot be underestimated.

This study has limitations. Firstly, all participants were based in urban melanoma centres, limiting the breadth of views and not reflecting physicians and nurses at regional centres. Secondly, when completing most interviews, adjuvant nivolumab or pembrolizumab was not subsidised by the Australian Government for resected stage III melanoma. However, many participants noted that patients accessed adjuvant immunotherapy drugs via compassionate access schemes or clinical trials. Thirdly, immunotherapy is one but not the only treatment strategy for resected stage III melanoma. Nonetheless, factors affecting recommendations for BRAF-targeted therapy were not the focus of this study.

Overall, this study provides insight into the factors physicians and nurses considered and the theoretical or qualitative treatment trade-offs they were willing to make when recommending immunotherapy. Further work is planned to quantify physician preferences for adjuvant immunotherapy. Understanding physician and nurse trade-offs highlights differences between physician and patient preferences for adjuvant immunotherapy, assisting health care professionals and patient communication regarding management options, and augmenting patient treatment decision-making. Integrating patient-reported outcome and experience measures into clinical practice may encourage discussion of factors other than the likelihood of recurrence that may be essential to a patient’s overall well-being.

## Conclusions

Physicians and nurses identified that clinical and patient factors such as melanoma sub-stage and the risk/benefit profile were primary considerations when recommending adjuvant immunotherapy to patients with resected stage III melanoma. Secondary factors included uncertainties about the effectiveness of adjuvant immunotherapy and not knowing which patients might benefit most from treatment. Additionally, participants considered what treatment burdens might be acceptable to their patients. Findings clarify clinician preferences and values, aiding clinical communication with patients and facilitating clinical decision-making about management options for resected stage III melanoma.

## Supplementary Information

**Additional file 1.**

**Additional file 2.**

## Data Availability

The datasets used and/or analysed during the current study are available from the corresponding author on reasonable request.
