# Supplementary material for: Adjuvant immunotherapy recommendations for stage III melanoma: physician and nurse interviews
Source: BMC Cancer. 2021 Sep 10;21:1014. doi: 10.1186/s12885-021-08752-1 (PMC8434723; doi:10.1186/s12885-021-08752-1)
Supplement: Supplementary file 1 — Additional file 1. [file 12885_2021_8752_MOESM1_ESM.docx]

# ADDITIONAL FILES

**ADDITIONAL FILE 1**

The MELT study – A focus group study of patients with stage 3 cutaneous MELanoma eligible to receive adjuvant immunoTherapy treatment and qualitative interviews with carers and treating clinicians.

Semi-structured interview guide and background questions for treating **clinicians** exploring preferences for treatment of patients with stage II-III melanoma eligible for adjuvant immunotherapy.

**Interview topic guide**

What is your role, and how do you discuss adjuvant immunotherapy as a treatment option for stage III melanoma patients?

And your stage II patients?

*Interviewer prompt (if needed): How is this conversation continued with the patient and their family by yourself and other healthcare professionals?*

Who do you think should be prioritised for adjuvant immunotherapy treatment?

*Interviewer prompt (if needed): For example, do factors like age, pts financial status, the stage of disease, location (rural/remote) have a role? If so, how?*

What factors do you think are important to patients and their families when making adjuvant immunotherapy treatment decisions?

What do you believe are the advantages of adjuvant immunotherapy treatment in this population?

Disadvantages?

*Interviewer prompt (if needed): What are your treatment goals?*

How do you resolve an issue if a patient makes a decision that conflicts with what you think is the optimal decision?

*Interviewer prompt: Has this ever happened? Can you describe an example?*

**For those patients receiving adjuvant immunotherapy**

Why do you think patients consider stopping or stop their immunotherapy treatment?

When a patient's outcome is not what you anticipated when on adjuvant immunotherapy treatment, for example, they have a recurrence and significant treatment toxicities, how does this make you feel?

How does that impact future patients in your care?

*Interviewer prompt: Has this ever happened? Can you describe an example?*

Will this treatment cost the patient and/or their family money? *Interviewer prompt (if needed): Can you tell me more about this? Were there any out of pocket costs, for example, medicine costs or gap payments for medicines, costs to attend treatment visits such as travel costs/parking fees, time off work for you or a family member or friend, travel time?*

Is there anything else that you think is important to add?

**Background questions**

What is your age?

What is your sex? Please tick one box:

Male □

Female □

Prefer not to answer □

Other (please specify)_______________________ □

What is your healthcare speciality?

Medical oncologist □

Surgeon □

Dermatologist □

Nurse □ Specify classification (CNS, CNC, RN, EN)

Other, specify_________________________

Do you work full or part-time?

Full time □

Part-time □

How many hours a week do you typically work?

How many years ago did you complete your healthcare training?

How many years ago did you complete your advanced/specialist training?

Location of melanoma clinic(s) located (metro, rural, regional)?

What percentage of patients you see have melanoma?

How many years of experience do you have managing patients with melanoma?

On average, how many patients do you see a month who are eligible for adjuvant immunotherapy?
